# Supplementary material for: A Critical View of the Application of the APEX Software (Aqueous Photochemistry of Environmentally-Occurring Xenobiotics) to Predict Photoreaction Kinetics in Surface Freshwaters
Source: Molecules. 2019 Dec 18;25(1):9. doi: 10.3390/molecules25010009 (PMC7017383; doi:10.3390/molecules25010009)
Supplement: Supplementary file 1 [file molecules-25-00009-s001.zip › 3.2.4_gcc-4.4.0.zip!/3.2.4_gcc-4.4.0/3.2.4_gcc-4.4.0/doc/octave/html/interpreter/Expressions.html]

Expressions - Untitled


Next: Evaluation,
Previous: Variables,
Up: Top


---

## 8 Expressions

Expressions are the basic building block of statements in Octave. An
expression evaluates to a value, which you can print, test, store in a
variable, pass to a function, or assign a new value to a variable with
an assignment operator.

An expression can serve as a statement on its own. Most other kinds of
statements contain one or more expressions which specify data to be
operated on. As in other languages, expressions in Octave include
variables, array references, constants, and function calls, as well as
combinations of these with various operators.

- Index Expressions- Calling Functions- Arithmetic Ops- Comparison Ops- Boolean Expressions- Assignment Ops- Increment Ops- Operator Precedence
